# Angiopoietin-2 and Angiopoietin-2/Angiopoietin-1 Ratio as Indicators of Potential Severity of *Plasmodium vivax* Malaria in Patients with Thrombocytopenia

**DOI:** 10.1371/journal.pone.0109246

**Published:** 2014-10-02

**Authors:** Luciano Teixeira Gomes, Eduardo Rodrigues Alves-Junior, Clebson Rodrigues-Jesus, Andreia Ferreira Nery, Thamires Oliveira Gasquez-Martin, Cor Jesus Fontes

**Affiliations:** 1 Hospital Julio Müller, Federal University of Mato Grosso, Cuiabá, Brazil; 2 Univag University Centre, Varzea Grande, Brazil; 3 Facimed Cacoal Faculty of Medicine, Cacoal, Brazil; Instituto de Ciências Biomédicas / Universidade de São Paulo - USP, Brazil

## Abstract

**Introduction:**

Angiogenic factors such as angiopoietin 1 (Ang-1) and angiopoietin 2 (Ang-2) are biomarkers produced during activation and dysfunction of the vascular endothelium in several infectious diseases. The aim of this study was to determine the serum levels of Ang-1 and Ang-2 and to establish their relationship with the main indicators of worst-case prognosis in patients with *P. vivax* malaria.

**Methods:**

This is a retrospective case-control study nested within a cohort of symptomatic malaria patients. A potentially severe case was defined as a patient that presented at least one of the main indicators of the worst-case prognosis for falciparum malaria, as established by the World Health Organization. Ang-2 and Ang-1 and the Ang-2/Ang-1 ratio were used to analyze the role of angiopoietins as biomarkers in signaling potentially severe vivax malaria. ROC curves were generated to identify a cut-off point discriminating between the angiopoietin concentrations that were most strongly associated with potential infection severity.

**Results:**

The serum levels of Ang-2 and the Ang-2/Ang-1 ratio were higher in the case group. In contrast, the serum levels of Ang-1 were lower in the cases than in the control patients. The blood count for platelets showed a positive correlation with Ang-1 and a negative correlation with Ang-2 and with the Ang-2/Ang-1 ratio. The area under the ROC curve (AUC) for serum angiopoietins, as an indicator of worst-case prognosis in a potentially severe *P. vivax* malarial infection, was larger in the subgroup of patients with platelet counts <75,000/µL.

**Conclusion:**

This study showed that patients with predictors of worst-case prognoses for *P. vivax* malaria have lower Ang-1 and higher Ang-2 serum levels (and higher values for the Ang-2/Ang-1 ratio) than controls. Elevated serum levels of Ang-2 and high values for the Ang-2/Ang-1 ratio may potentially be used as predictors of worst-case prognoses for *P. vivax* malaria, especially in patients with thrombocytopenia.

## Introduction

Over the past few years, the number of publications on severe *Plasmodium vivax* malaria has increased, moving away from the conventional idea that this infection is benign [1], [2]. In several studies of patients diagnosed with severe *P. vivax* malaria, multiple syndromes have been described, similar to those caused by *P. falciparum* [3]. Although similar, the clinical symptoms are not the same nor of the same intensity. However, researchers have begun to use the same criteria to define the severity of malaria caused by *P. vivax* as those proposed by the World Health Organization [4] to define the severity of malaria caused by *P. falciparum* [5], [6].

Compared to *P. falciparum*, *P. vivax* has a greater capacity to induce an inflammatory response, which results in a lower pyrogenic threshold [7]. However, the typical pathogenic mechanisms of cytoadherence and microvascular sequestration of parasites, present in severe infections caused by *P. falciparum*, are less frequent in severe vivax malaria [2], [3]. There is evidence that links worst-case prognosis criteria for severe malaria caused by *P. falciparum* and severe infections caused by *P. vivax*, of which severe anemia [8], acute pulmonary edema [6], coma [9], [10], acute kidney injury [11], jaundice [11], , and shock [13] are already known.

A number of laboratory markers have been used to assess inflammatory patterns in response to malaria and their relation to the severity of the infection [12]. Biomarkers for inflammation as a means of monitoring the clinical progress of patients infected with *P. vivax* have been described, including those for endothelial lesions, hemolysis, and oxidative stress. Numerous substances have been evaluated as biomarkers for the severity of malarial infections such as proteins associated with iron metabolism and anti-oxidant enzymes, as well as various cytokines and chemokines [14], [15], [16]. Although not considered to be a criterion for severe malarial infection, thrombocytopenia has been frequently reported in *P. vivax* infections, and its association with severe cases has been established [12], [17].

An understanding of the relationship between the presentation of the disease and the production of a biomarker may help in clinical decision-making and could minimize the risk of disease complications and consequently, the suffering of patients [16]. Angiogenic factors such as angiopoietin 1 (Ang-1) and angiopoietin 2 (Ang-2) are biomarkers produced during activation and dysfunction of the vascular endothelium in non-infectious as well as infectious diseases, including malaria [18], [19]. Ang-1 and Ang-2 are antagonistic ligands of the Tie-2 receptor that is expressed on the surface of the vascular endothelium. In healthy individuals, the levels of Ang-1 are higher than those of Ang-2, thus, promoting the stability of the endothelium and preventing the activation of a proinflammatory response [20]. Inflammation, however, promotes the liberation of Ang-2, and the increased binding of the Tie-2 receptors with Ang-2 induces the generation of proinflammatory and prothrombotic responses [21].

Recent studies have elucidated the relationship between disease severity and plasma levels of angiopoietins in *P. falciparum* infections, as well as that between the levels of these proteins and the occurrence of cerebral malaria, placental malaria, retinopathy, and death [22], [23], [24], [25]. However, studies on angiopoietins in *P. vivax* infections [26], [27], as well as on associations between this biomarker and the occurrence of clinical and laboratory evidence for severity, are limited. The aim of this study was to determine the serum levels of angiopoietins (Ang-1 and Ang-2) and to establish their relationship with the main indicators of a worst-case prognosis in patients with *P. vivax* malaria.

## Methods

### Patients and method

This retrospective case-control study was performed within a cohort of malaria patients treated at the Hospital Julio Müller, in the city of Cuiabá, state of Mato Grosso, Brazil, between December 2011 and November 2013. The hospital is a reference center for the diagnosis and treatment of malaria in people living and traveling in the south and southeast of the Brazilian Amazon. Participation in the study was voluntary, with a free and informed consent form signed by the patient, or in the case of children, by the parents. This study was approved by the Ethics Committee of the Julio Müller School Hospital (Document 130/CEP/HUJM/2011).

Patients with symptomatic malaria were eligible to participate in the study if they had a confirmed monoinfection with *P. vivax*, as confirmed by microscopy of a thin blood smear and polymerase chain reaction (PCR) analysis. Following the confirmation of the diagnosis, the patients underwent clinical examination, as well as hematology and blood biochemistry tests, including those for serum levels of Ang-1 and Ang-2. To analyze the role of angiopoietin as a biomarker for potentially severe vivax malarial infections, a subgroup of patients were classified as potentially severe cases, if they presented at least one of the main indicators (for children and adults) of the worst-case prognosis for falciparum malaria, as established by the World Health Organization [4]. The defining indicators were as follows: hemoglobin concentration <5 g/dL; erythrocyte volume fraction <15%; serum creatinine >3 mg/dL; blood urea >60 mg/dL; blood glucose <40 mg/dL; total bilirubin >2.5 mg/dL; greater than threefold elevation in serum transaminases; parasitemia >250,000/µL. Control group patients, who did not present any of these indicators, were randomly selected.

### Sample Collection and Storage

Ang-1 is present in platelet granules and is released upon platelet activation [28]. Therefore, to measure circulating levels of Ang-1, platelet-free serum should be collected for measurement. Five milliliters of blood were collected into serum-separating tubes and allowed to clot at room temperature for 30 min before centrifugation for 15 min at 1000×*g*. The serum was removed and aliquots were stored at −80°C. The serum was defrosted only once prior to the assay for angiopoietin.

### Laboratory analyses

The hematological evaluation of the patients was performed using a Cell Dyn Ruby multi-parameter automated hematology analyzer (Abbott Laboratories, Illinois, USA). Blood biochemistry was analyzed by photometry using a BT-3000 Plus automated chemistry analyzer (Diamond Diagnostics, Massachusetts, USA).

Angiopoietin levels in the serum samples were assessed by enzyme-linked immunosorbent assay (ELISA) using standard commercial kits (R&D Systems, Minneapolis, USA) for Ang-1, the Quantikine Angiopoietin-1 kit (Lot 306702), and the Quantikine ELISA Human Angiopoietin-2 kit (Lot 308323) for Ang-2, according to the manufacturer’s instructions.

### Statistical analysis

Because of the non-normality of the data, non-parametric statistics were used for the analysis. The Mann–Whitney *U* test was used to compare the distribution of the classic indicators for potentially severe malarial infection between the cases and the control patients. Spearman’s correlation test was used to analyze the association between angiopoietins and platelet counts. Because of heteroscedasticity, we used the non-parametric Kruskal–Wallis test to compare the values of the levels of angiopoietin and classified the patients into three groups according to their platelet counts: severe thrombocytopenia (<50,000/µL), moderate thrombocytopenia (50,000 to 150,000/µL), and no thrombocytopenia (>150,000/µL). Cuzick’s test for trend was used to analyze the tendency of quantitative variables between platelet counts of the different groups analyzed. We generated receiver operating characteristic (ROC) curves and calculated the area under the ROC curve (AUC) to evaluate the diagnostic accuracy of angiopoietins for discriminating between patients with and without the indicators of the worst-case prognosis for potentially severe vivax malaria. The Youden ‘J’ index was used to define the cut-off points corresponding to the sensitivity and specificity values with the lowest probability of having occurred randomly [29]. P-values<0.05 were considered significant for all tests.

## Results

Eighty patients with symptomatic malaria caused by PCR-confirmed *P. vivax* monoinfection were included in the study. Of the 80 patients, 58 (72.5%) were men and 22 (27.5%) were women, with ages ranging from 5 to 78 years, with a median (1^st^–3^rd^ quartile) of 39 (26–49) years. All had presented with fever within the 24 h prior to entering the study. Altered hematologic and biochemical parameters were infrequent except for platelet counts, for which the median (1^st^–3^rd^ quartile) was 98,000 (62,500–168,000) platelets/µL for all patients. Severe and moderate thrombocytopenia were observed in 10 (12.5%) and 45 (56.2%) patients, respectively. Only 25 (31.3%) of the patients showed normal platelet counts. The clinical and laboratory characteristics of patients according to platelet count are shown in Table 1. Only total serum bilirubin and urea levels tended to decrease as the platelet count increased (P = 0.006 and P = 0.015, respectively).

**Table 1:** Baseline clinical and laboratory characteristics of the patients in each platelet group.

Characteristics	Platelet groups (Median)			p*
	<50,000	50,000 to 150,000	>150,000	
Age (years)	47.5	38.0	38.0	0.435
Hemoglobin (g/dL)	13.0	12.9	12.1	0.488
Hematocrit (%)	38.0	37.9	35.6	0.367
Bilirubin (mg/dL)	1.7	1.3	0.9	0.006
Urea (mg/dL)	41.5	30.0	28.0	0.015
Creatinine (mg/dL)	1.1	0.9	0.9	0.159
Aspartate aminotransferase (U/L)	23.5	28.0	21.0	0.288
Alanine aminotransferase (U/L)	26.5	29.0	29.0	0.952
Parasitemia (/µL)	8,600	6,160	5,000	0.524
Axillary temperature (°C)	36.7	37.0	36.4	0.610

*Cuzick’s test.

Eighteen patients were classified as potentially severe cases in this study, showing one or more of the main indicators of the worst-case prognosis for malaria: severe anemia (n = 1), elevated blood urea nitrogen (BUN) (n = 3), hyperbilirubinemia (n = 10), and elevated serum transaminases (n = 4). The ages of the potentially severe cases did not differ from those of the controls (P = 0.870). As expected, however, the parameters signaling severe malarial infection were altered in the potentially severe cases, with significant differences in serum bilirubin levels (P = 0.008), aspartate aminotransferase (AST) (P<0.001), alanine aminotransferase (ALT) (P = 0.002), and parasitemia (0.010). No significant differences in hemoglobin (P = 0.760), hematocrit (0.471), serum urea (0.179), serum creatinine (P = 0.061), platelet count (P = 0.097), or axillary temperature (P = 0.126) were observed between cases and controls (Table 2).

**Table 2:** Baseline clinical and laboratory characteristics of patients that were used in defining a severe *Plasmodium vivax* infection.

Characteristics	Case n = 18 median (1^st^–3^rd^ quartile)	Control n = 62 median (1^st^–3^rd^ quartile)	p*
Age (years)	38.5 (25–55)	39.0 (28–49)	0.870
Hemoglobin (g/dL)	12.8 (12.6–14.0)	12.9 (11.1–14.3)	0.760
Hematocrit (%)	37.8 (37.0–41.2)	37.2 (32.8–41.8)	0.471
Bilirubin (mg/dL)	2.8 (1.9–3.4)	1.1 (0.8–1.4)	0.008
Urea (mg/dL)	34 (19–41)	29 (25–34)	0.179
Creatinine (mg/dL)	1.0 (0.9–1.2)	0.9 (0.8–1.0)	0.061
Aspartate aminotransferase (U/L)	54 (26–76)	22 (17–30)	<0.001
Alanine aminotransferase (U/L)	45 (31–104)	25.5 (16–37)	0.002
Parasitemia (/µL)	11,625 (7,200–17,500)	4,500 (1,900–9,500)	0.010
Axillary temperature (°C)	37.7 (36.6–39.0)	36.4 (36.0–38.0)	0.126
Platelet count (/µL)	78,500 (55,000–110,000)	113,000 (64,000–175,000)	0.097
Angiopoietin-1(ng/mL)	12 (9.8–17.6)	19.9 (13.1–29.2)	0.005
Angiopoietin-2 (ng/mL)	8.2 (5.9–14.8)	5.9 (3.9–7.9)	0.032
Angiopoietin-2/Angiopoietin-1 ratio	0.8 (0.3–1.5)	0.3 (0.2–0.6)	0.002

*Mann–Whitney *U* test.

**Figure 1:**
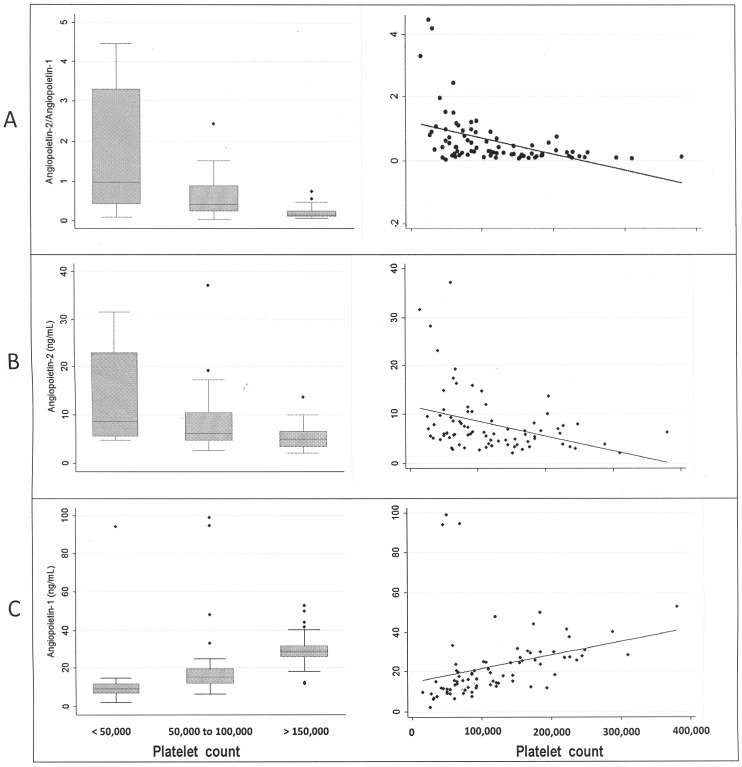
Boxplot and scatter graphs showing the association between the angiopoietin-2/angiopoietin-1 ratio (A), levels of angiopoietin-2 (B), and levels of angiopoietin-1 (C) with platelet counts in *P. vivax* malaria patients.

Angiopoietin levels were not associated with other factors known to be related to the severity of malaria, such as age, level of parasitemia, or axillary temperature. When the analysis of the serum levels of angiopoietin was stratified according to the patients’ platelet counts, we found that the median value of Ang-1 was lower (P = 0.0001) in the patients with thrombocytopenia than in those with normal platelet counts. The median values for Ang-2 and the Ang-2/Ang-1 ratio, on the other hand, were higher (P = 0.012 and P = 0.0001, respectively) in the patients with thrombocytopenia. As the platelet count increased, so did the levels of Ang-1 (P<0.001) but the levels of Ang-2 decreased (P = 0.003) as did the Ang-2/Ang-1 ratio (P<0.001). In summary, regardless of whether or not the patient met the worst-case prognosis criteria, the blood count for platelets showed a positive correlation with Ang-1 (r = 0.6; P<0.001) and a negative correlation with Ang-2 (r = −0.4; P = 0.001) and with the Ang-2/Ang-1 ratio (r = −0.6; P<0.0001) (Figure 1).

**Figure 2:**
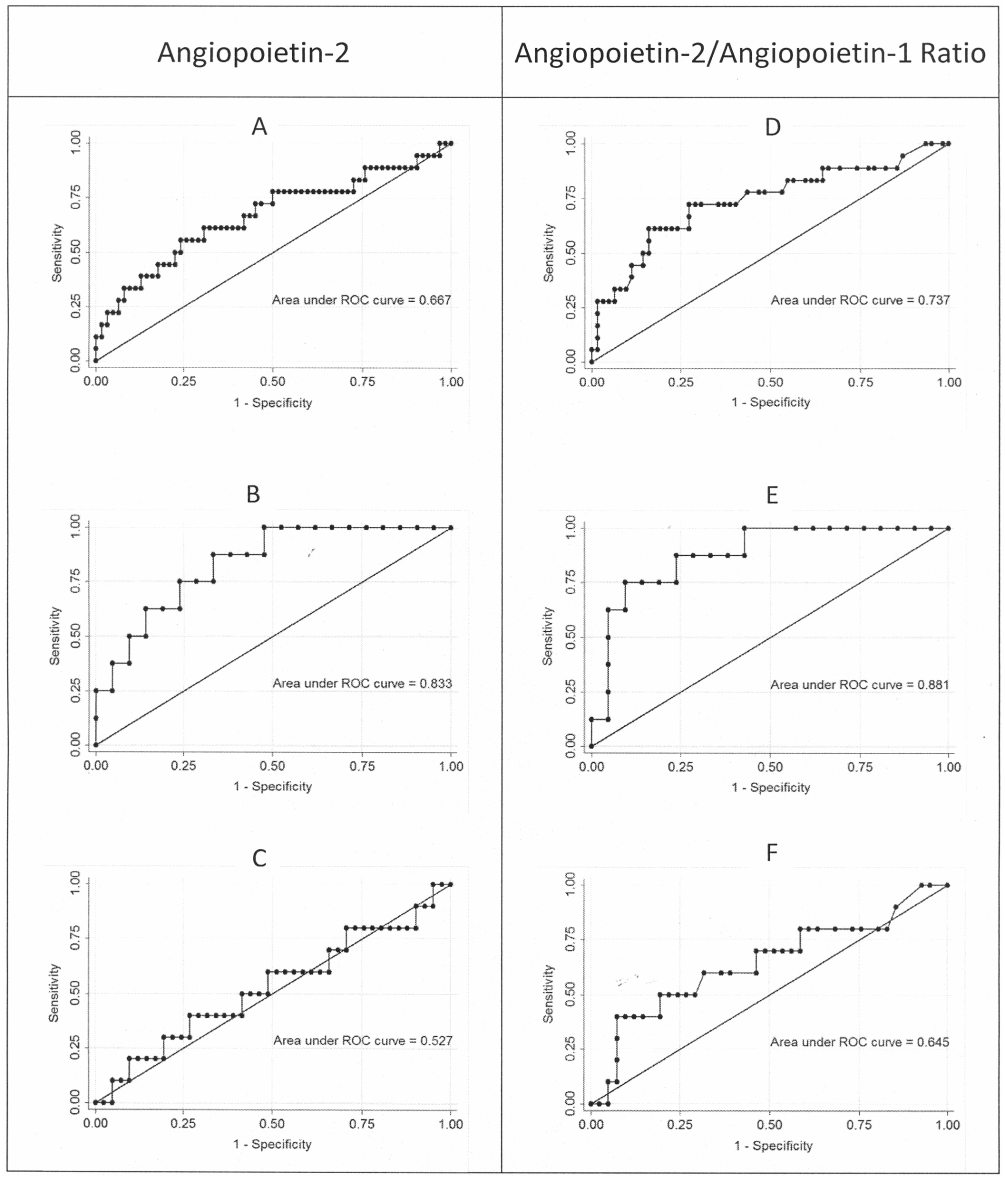
Assessment of the utility of Ang-2 levels and the Ang-2/Ang-1 ratio in discriminating between potentially severe malaria (cases) and controls using ROC analysis for all patients (A, B), patients with platelet counts <75,000/µL (C, D), and patients with platelet counts >75,000/µL (E, F). The reference line represents the ROC curve for a test with no discriminatory ability. The area under the ROC curve (AUC) is displayed on each graph.

ROC curves were generated to identify a cut-off point to discriminate between angiopoietin concentrations that were most strongly associated with the potential severity of the case, including the subgroups of patients stratified according to the platelet blood count. From the ROC curve, a highly accurate cut-off level for serum angiopoietins was defined as an indicator for a worst-case prognosis of a potentially severe malarial infection. In the analysis of all patients, the AUC was 0.667 and the cut-off point for Ang-2 was 5.9 ng/mL (77.8% sensitivity and 50.0% specificity), whereas for the Ang-2/Ang-1 ratio, the AUC was 0.737 and the cut-off point was 0.75 (61.1% sensitivity and 83.9% specificity). Considering only the subgroup of patients with platelet counts <75,000/µL, the ROC curve showed an AUC of 0.833 and a cut-off point of 8.4 ng/mL for Ang-2 (87.5% sensitivity and 66.7% specificity), whereas for Ang-2/Ang-1, the AUC was 0.881, with a cut-off point of 1.2 (75.0% sensitivity and 90.5% specificity). In patients with platelet counts >75.000/µL, the AUC was 0.527 and the cut-off point was 3.85 ng/mL for Ang-2 (80.0% sensitivity and 29.3% specificity), whereas for Ang-2/Ang-1, the AUC was 0.645 and the cut-off point was 0.30 (60.0% sensitivity and 68.3% specificity) (Figure 2).

## Discussion

In this study, low levels of Ang-1 and high values of Ang-2 and the Ang-2/Ang-1 ratio were associated with criteria that indicate the worst-case prognosis in patients with symptomatic *P. vivax* infections. In *P. falciparum* malaria, the production of angiogenic factors is principally associated with an increase in the cytoadherence of infected erythrocytes to the vascular endothelium [18]. Previous studies have demonstrated that *P. vivax* promotes cytoadhesion [30], [31]. However, the mechanisms involved in endothelial activation and vascular alterations associated with *P. vivax* infections remain unclear. Only two studies have described the relationship between angiopoietins and malaria caused by *P. vivax*, and these presented conflicting results. One report showed lower levels of Ang-2 in patients infected by *P. vivax* than in patients infected by *P. falciparum* [27], whereas the other report detected higher levels of Ang-2 in patients infected by *P. vivax* [26]. However, no information is currently available on levels of Ang-1 or the Ang-2/Ang-1 ratio.

Angiopoietins are proteins that are intimately involved in the stability of the endothelium [21] and their levels are altered in various inflammatory conditions, mainly due to endothelial activation [19] in malarial infections and other infectious diseases [32]. Under normal conditions, higher levels of Ang-1 promote quiescence in the vascular endothelium. In inflamed areas, however, Ang-2 levels are higher, principally in the endothelial cells and the smooth muscles, thus activating the vascular endothelium. *In vitro* and *in vivo* studies have demonstrated that higher concentrations of Ang-2 are associated with elevated production of TNF-α, nitric oxide, vascular endothelial growth factor (VEGF), and hypoxia [33], [34].

In this study, a positive correlation was observed between platelet counts and Ang-1 levels in *P. vivax* malaria patients, whereas a negative correlation was detected between platelet counts and Ang-2 and Ang-2/Ang-1 levels. Although not a defining feature of severe malaria, vivax-associated thrombocytopenia occurs in more than 24% of patients with vivax malaria [35], [36]. Its mechanism is multifactorial; it has been associated with oxidative stress, alterations in splenic function, invasion of the bone marrow by trophozoites, and destruction by immunoglobulins [36], [37]. Vivax-related thrombocytopenia has been associated with severe manifestations [11], [12] and the need for blood and platelet transfusions was reported in 5% of adults with severe vivax malaria [11].

The association between Ang-1, Ang-2, and the Ang-2/Ang-1 ratio was stronger in patients with thrombocytopenia. Some possible explanations for this association are given below. Firstly, Ang-1 is secreted by platelet granules, and thus its concentration in the plasma is dependent on the number of circulating platelets [38]. Our results support the findings of Browers et al. (2013), who detected elevated serum Ang-1 levels following the recovery of platelet numbers in patients treated for falciparum malaria [39]. Secondly, a prior study involving patients with vivax and falciparum malaria has shown increased thrombogenic activity and a reduction in platelet count associated with high production of cytokines and microparticles, and consequently, endothelial activation and an increase in angiogenic factors in the serum [40], [41], [42]. The observation of higher levels of Ang-2 and Ang-2/Ang-1 in patients with thrombocytopenia in this study supports these findings and validates the association between thrombocytopenia caused by *P. vivax* and the elevation of Ang-2 levels.

Although useful for clinical decision-making, biomarkers that predict the appearance of criteria indicating the worst-case prognosis for *P. vivax* malaria patients have not been identified to date. Disequilibrium in the expression and production of angiogenic factors is associated with the severity of malaria caused by *P. falciparum* [22], [18], [43], [44]. It has also been found that Ang-2 is a better prognostic marker to signal imminent severity in *P. falciparum* malaria than lactate [18], which is the traditional laboratory marker for severity [4]. High plasma levels of Ang-2 have also been associated with worst-case prognoses for other diseases such as leukemia, metastatic melanoma, acute pulmonary damage, and acute hepatic and renal failure [19]. To summarize, Ang-2 and Ang-2/Ang-1 have been considered as predictive biomarkers for the severity of various pathologies, both non-infectious [45], [46], [47] and infectious [32], [48], including falciparum malaria [15], [23], [49], [50]. The Ang-2/Ang-1 ratio has already been considered as a more effective biomarker of severity than Ang-2 alone in falciparum malaria [50], as well as in other diseases [51], [52].

To the best of our knowledge, this study provides the first evidence of the relationship between changes in angiopoietin levels and criteria indicating the worst-case prognosis for *P. vivax* malaria, especially in patients with low platelet counts. Once incorporated into routine hospital laboratory investigation, the systematic assay of angiopoietins could help isolate patients with the potential for developing severe malaria during clinical management of vivax malaria.

The main limitation of this study is the small number of patients examined, thus restricting the possibilities for extrapolating the results to other populations. It is also important to point out that the comorbidities of the patients studied may explain the findings suggesting a worst-case prognosis, as already shown in the literature [6]. However, in this study it was possible to exclude some acute morbidities, such as dengue, yellow fever, and types of viral hepatitis, leptospirosis, bacterial infections, pneumonia, heart failure, hepatic cirrhosis, and diabetes mellitus. However, further studies involving a larger number of patients and more research into comorbidities should be performed to validate our findings.

## Conclusions

This study showed that patients with worst-case prognoses for *P. vivax* malarial infections have lower serum Ang-1 and higher serum Ang-2 levels (and higher values for the Ang-2/Ang-1 ratio) than controls. Furthermore, a positive correlation was observed between platelet count and Ang-1, and a negative correlation was observed between platelet count and Ang-2 (and the Ang-2/Ang-1 ratio). Elevated serum levels of Ang-2 and high values for the Ang-2/Ang-1 ratio may potentially be used as predictors of worst-case prognoses for *P. vivax* malaria, especially in patients with thrombocytopenia.
