# Correction: Angiopoietin-2 and Angiopoietin-2/Angiopoietin-1 Ratio as Indicators of Potential Severity of *Plasmodium vivax* Malaria in Patients with Thrombocytopenia

**DOI:** 10.1371/journal.pone.0117651

**Published:** 2015-01-28

**Authors:** The PLOS ONE Staff

There are a number of errors in the legend for Fig. 2, “Assessment of the utility of Ang-2 levels and the Ang-2/Ang-1 ratio in discriminating between potentially severe malaria (cases) and controls using ROC analysis for all patients (A, B), patients with platelet counts <75,000/μL (C, D), and patients with platelet counts >75,000/μL (E, F).” The complete, correct Fig. 2 legend is:

**Figure 2:**
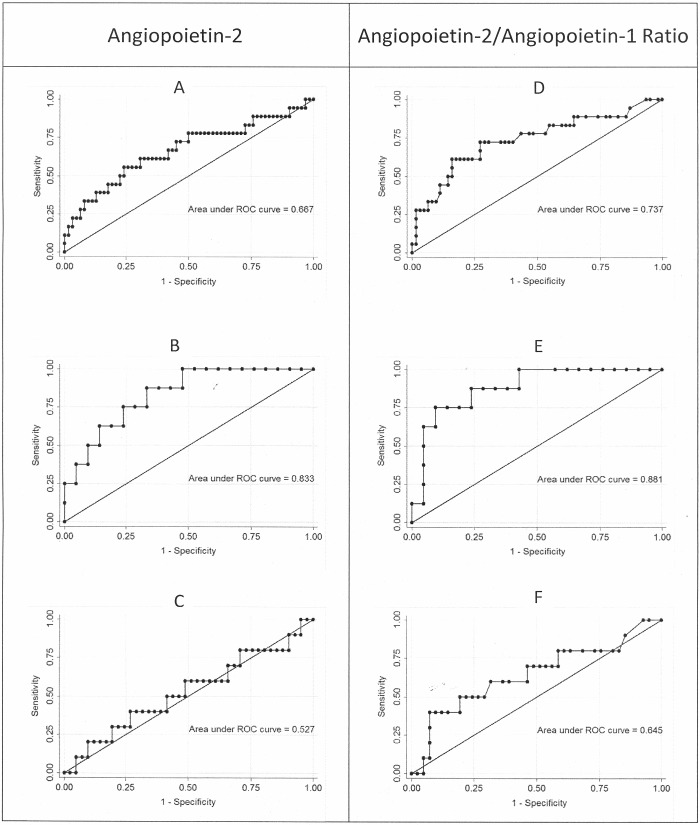
Assessment of the utility of Ang-2 levels and the Ang-2/Ang-1 ratio in discriminating between potentially severe malaria (cases) and controls using ROC analysis for all patients (A, D), patients with platelet counts <75,000/mL (B, E), and patients with platelet counts >75,000/mL (C, F). The reference line represents the ROC curve for a test with no discriminatory ability. The area under the ROC curve (AUC) is displayed on each graph.
